# Information non précise sur la taille du pénis en République Démocratique du Congo: à propos de 21 sources

**DOI:** 10.11604/pamj.2016.24.291.9945

**Published:** 2016-07-29

**Authors:** Philippe Cilundika Mulenga, Alex Bukasa Kazadi

**Affiliations:** 1 Département de Santé Publique, Faculté de Médecine, Université de Lubumbashi, République Démocratique du Congo; 2 Service de Sexologie Clinique, Centre Hospitalier Elohim, République Démocratique du Congo

**Keywords:** Information précise, taille du pénis, République Démocratique du Congo, RD Congo, RDC, Accurate information, penis size, Democratic Republic of the Congo

## Abstract

**Introduction:**

La taille du pénis constitue une préoccupation de beaucoup des gens actuellement et certains ne sont pas satisfaits de la dimension de leur pénis comme le montre l’étude de Tiggemann en 2008. Il existe relativement peu d'études sur le pénis en érection. Cela peut refléter les tabous culturels des chercheurs ou des médecins en interaction avec les hommes qui sont dans un état d’excitation sexuelle. Toutes fois, il est important pour les personnes qui annoncent des détails sur la taille du pénis d’annoncer d’abord les repères de la mesure du pénis puis ensuite donner les chiffres que proposent les chercheurs.

**Méthodes:**

Notre enquête de type descriptif transversale s’est effectuée dans les deux grandes villes de la République Démocratique du Congo à savoir la ville de Kinshasa et la ville de Lubumbashi, pendant une période de deux ans soit de Mai 2014 à Mai 2016. Au total, 21 sources d’information ont constitué notre échantillon dont 8 à Kinshasa et 13 à Lubumbashi et nous avons trouvé cela suffisant car les sujets à caractère sexuel sont souvent rares chez nous. Les paramètres étudiés étaient: la nature de la source, la précision de la technique de la mesure, la présence de référence bibliographique, la longueur annoncée du pénis.

**Résultats:**

La majorité des sources d’information sont faites des émissions de radio et de télévision (23,8%), ceci pourra s’expliquer par le fait que dans notre milieu il y a de plus en plus des chaines de radio et télévision et surtout dans les grandes villes. Concernant la précision de la technique de la mesure du pénis lors du partage du message sur la taille du pénis, l’étude nous montre que la majorité des sources d’information ne signale pas cela lorsqu’elles annoncent la taille du pénis au public soit 85,7%. Plusieurs sources ne déclarent pas les références bibliographiques (57,1%). Lorsqu’on regarde même les chiffres de la taille du pénis annoncée, l’on voit les proportions majoritaires suivantes: 14 cm comme taille normale soit 28,6%, suivi de ceux là qui disent 15 cm soit 23,8% et de 15 à 20 cm soit 19%.

**Conclusion:**

Ces résultats sont une interpellation des tous les acteurs responsables de la diffusion de l’information sur la santé sexuelle (taille du pénis): la rigueur scientifique consiste à chercher l’information dans des sources fiables.

## Abstract (en)

**Introduction:**

Penis size is a huge topic of anxiety for a lot of men. Some of them are unhappy with their penis size as shown in the study conducted by Tiggemann in 2008. There are relatively few studies on erect penis size. This may reflect cultural taboos of researchers or doctors interacting with men who are in a state of sexual arousal. On the other hand, it is important for people who announce details on penis size to give the average penis size first and then sizes suggested by the researchers.

**Methods:**

We performed a cross-sectional survey in the two major urban centres of the Democratic Republic of Congo namely Kinshasa and Lubumbashi over a period of two years from May 2014 to May 2016. A total of 21 information sources constituted our sample, 8 in Kinshasa and 13 in Lubumbashi. We found it sufficient because in our culture discussing about sexual matter is rare. The parameters studied were: the nature of the source, the accuracy of the measurement method, the presence of bibliographical reference, the announced penis size.

**Results:**

The majority of information sources used were radio or television broadcastings (23,8%); this can be explained by the fact that there are an increasing number of radio and television stations in our country and especially in large cities. With regard to accuracy of information about penis measurement method when sharing the message about penis size, our study showed that the majority of information sources did not indicate it when they announced penis size to the public (85,7%). Several sources did not report bibliographical references (57,1%). Announced data analysis on penis size showed that the average penis size was: 14 cm (28,6%), 15 cm (23,8%) and 15-20 cm (19%).

**Conclusion:**

All these results are intended to offer a warning to all players responsible for diffusing information on sexual health (penis size): scientific rigor consists in seeking information from reliable sources.

## Introduction

La taille du pénis constitue une préoccupation de beaucoup des gens actuellement et certains ne sont pas satisfaits de la dimension de leur pénis comme le montre l’étude de Tiggemann en 2008 [1]. Les données disant que la taille du pénis était liée à la taille de la personne, ou à la taille des chaussures ou à l’age ou au régime de la personne ne sont pas fiables [2]. Les préoccupations relatives à la taille du pénis affectent la satisfaction et le fonctionnement sexuel des hommes [2]. Bien sûr, la taille du pénis ne doit pas affecter les fonctions sexuelles comme l´orgasme ou le désir sexuel [3]. Un plus petit pénis diminue la confiance sexuelle [4]. Concernant la mesure du pénis, beaucoup d’études de la longueur du pénis au repos et étiré sont caractérisées [5] par des mesures personnelles [6]. Il existe relativement peu d´études sur le pénis en érection. Cela peut refléter les tabous culturels des chercheurs ou des médecins en interaction avec les hommes qui sont dans un état d’excitation sexuelle [2]. Au fait, pour mesurer le pénis, il y a des spécificités et cela est important. Bien qu'il n'y ait pas de technique standard pour la mesure de la taille du pénis, il semble y avoir un consensus parmi les chercheurs que la longueur du pénis doit être mesurée sur la face dorsale du pénis en commençant de la jonction pubopenile à l'extrémité du gland [7]. Cette mesure s'applique au pénis dans trois états: flasque, étiré et en érection. En outre, pour la mesure de la circonférence du pénis, il faut le faire au milieu du corps du pénis, dans les trois états. Par souci de clarté de la nomenclature, un pénis flasque est celui qui est non stimulé ou non excité, et saurait être vu quand l'homme est dans la position anatomique normale. Flasque étiré est quand le pénis flasque est tiré à sa distance maximale. Enfin un pénis en érection est celui qui est stimulée au maximum, que ce soit par le biais visuel, tactile ou manipulation pharmaceutique [8]. Toutefois, il est important pour les personnes qui annoncent des détails sur la taille du pénis de connaitre d’abord les repères de la mesure du pénis puis ensuite d’annoncer les chiffres que proposent les chercheurs; ceci pourrait rassurer les personnes complexées et les adolescents de notre milieu qui sont aussi à la merci des images que proposent actuellement les médias et aussi les parents qui doivent intervenir à temps lorsqu’il remarque un micro pénis chez les enfants avant l’age 17 ans.

## Méthodes

Notre enquête de type descriptive transversale s’est effectuée dans les deux grandes villes de la République Démocratique du Congo à savoir la capitale Kinshasa et la deuxième ville du pays, Lubumbashi pendant une période de deux ans soit de Mai 2014 à Mai 2016. L’étude s’est faite auprès de sources d’information véhiculant un message sur la taille du pénis. Il est important de signaler que dans notre milieu, le sujet d’ordre sexuel est souvent tabou, ce qui explique le délai de deux ans pour l’enquête. Nous avons considéré comme source: les personnes (professionnels de santé, tradipraticiens ou phytothérapeutes) pouvant nous donner les détails sur le pénis de l’homme, les émissions de radio ou de télévision diffusant un message sur la taille du pénis, les cours donnés dans des facultés ou écoles, les livres publiés dans notre milieu et donnant les détails sur la taille du pénis. Toutes ces sources d’information sur la taille du pénis étaient les plus disponibles pendant cette période d’étude. Au total, 21 sources d’information ont constitué notre échantillon dont 8 à Kinshasa et 13 à Lubumbashi et nous avons trouvé cela suffisant car les sujets à caractère sexuel sont souvent rares chez nous. Les paramètres étudiés étaient: la nature de la source, la précision de la technique de la mesure, la présence de références bibliographiques, la longueur du pénis annoncé. Les données collectées étaient encodées, saisies, traitées et analysées à l’aide du logiciel Excel 2013. Et les résultats présentés sous forme des tableaux.

## Résultats

La grande proportion de la nature des sources d’information était constituée des chaines de Radio et télévision soit 23,8%, suivi des médecins généralistes (19%). La plus petite proportion de la nature des sources de l’information était constituée des spécialistes en Chirurgie, des infirmiers, des phytothérapeutes et d’un livre publié soit 4,7% (Tableau 1). Concernant la précision de la technique de mesure du pénis lors du partage du message sur la taille du pénis, l’étude nous montre que la majorité des sources d’information ne signale pas cela lorsqu’elles annoncent la taille du pénis au public soit 85,7%; les autres sources désignent soit le pubis comme repère de la mesure (4,7%), soit l’os pubien comme repère (4,7%) ou “connait les repères mais refuse de le dire” ( 4,7%) (Tableau 2). S’agissant de la présentation des références bibliographiques, 57,1% des sources d’information ne les signalent pas et les autres s’inspirent soit des cours reçus à l’école (28,6%) ou des notions apprises dans leurs spécialisations (14,3%) (Tableau 3). Lorsqu’on regarde même les chiffres de la taille annoncée du pénis, l’on voit les proportions majoritaires suivantes: 14 cm comme taille normale (28,6%), suivi de ceux là qui disent 15 cm (23,8%) et de 15 à 20 cm ( 19%) (Tableau 4).

**Tableau 1:** Distribution des sources d’information selon la nature

Nature de la source	Effectifs	Pourcentage
Spécialistes en sexologie	3	14,2
Spécialiste en Chirurgie	1	4,7
Médecins généralistes	4	19
Infirmiers	1	4,7
Tradipraticiens	2	9,5
Phytothérapeutes	1	4,7
Cours de Médecine	3	14,2
Chaine de radio et télévision	5	23,8
Livre publié	1	4,7
**Total**	**21**	**100**

**Tableau 2:** Distribution des sources d’information selon la précision de la technique de la mesure du pénis

Précision de la technique de mesure	Effectifs	Pourcentage
Sur l’os pubien	1	4,7
Sur le pubis	1	4,7
Connait mais refuse de donner	1	4,7
Aucune	18	85,7
**Total**	**21**	**100**

**Tableau 3:** Distribution des sources d’information selon les références bibliographiques

Références Bibliographiques	Effectifs	Pourcentage
D’après les études faites (spécialisation)	3	14,3
D’après les cours reçus à la faculté ou à l’école	6	28,6
Aucune référence déclarée	12	57,1
**Total**	**21**	**100**

**Tableau 4:** Distribution des sources d’information selon la longueur normale du pénis

Longueur déclarée du pénis normal	Effectifs	Pourcentage
15 à 20 cm	4	19
12 à 18 cm	2	9,5
14 à 18 cm	1	4,7
14 à 16 cm	1	4,7
12 à 15 cm	1	4,7
15 cm	5	23,8
14 cm	6	28,6
Pas de précision	1	4,7
**Total**	**21**	**100**

## Discussion

Nous constatons que la majorité des sources d’information sont faites des émissions de radio et de télévision (23,8%), ceci pourra s’expliquer par le fait que dans notre milieu il y a de plus en plus des chaines de radio et télévision et surtout dans les grandes villes; les animateurs trouvent des occasions pour parler de la santé sexuelle et très souvent dans les heures tardives pour garder davantage l’aspect tabou qui caractérise notre milieu. Très peu de livres sont écrits à ce sujet, la raison est peu être le manque de moyens ou juste le gène pour parler d’un tel sujet. Les médecins sexologues représentent une faible proportion comme source d'information et ils sont tous dans la capitale Kinshasa; ceci peut être du soit au manque des moyens des autres médecins pour entreprendre cette spécialisation ou alors au manque d'intérêt pour la sexologie. Hors ils doivent être des personnes-ressources pour répondre à toutes les inquiétudes de la communauté. Par rapport à l’annonce de la technique de la mesure du pénis, la plupart des sources n’expliquent pas la technique de mesure de la taille du pénis; ceci peut être du soit à une limitation à l’accès aux sources d'information fiable qui entraine une ignorance chez la plupart des gens et au fait de cacher la technique de mesure au public. Les chiffres sur la longeur du pénis peuvent aussi être intentionnellement exagérés afin d'attirer le public à l’agrandissement du pénis qui est en publicité un peu partout. Quant à la présence des références bibliographiques dans l’annonce des chiffres, il s’agit d’un grand problème: plusieurs sources ne déclarent pas de références bibliographique; plusieurs explications peuvent être attribuées à ce fait: l’ignorance et le désir de sensationnalisme; soit le manque de rigueur dans le montage des émissions ou dans la rédaction des livres publiés; tout travail scientifique doit avoir des références pour être validé; et même, les professionnels doivent chercher la vrai information à travers l’internet et les bibliothèques pourtant disponibles dans notre milieu. Lorsque nous voyons, les chiffres annoncés de la taille du pénis, on remarque une diversité importante, les mesures de 15 à 20 cm sont de très différentes de la norme annoncée par l’étude de Dr Veale [7], démontrant surement un manque de rigueur de la part de ceux là qui l’ont annoncé.

## Conclusion

Ces résultats sont une interpellation des tous les acteurs responsables de la diffusion de l’information sur la santé sexuelle: la rigueur scientifique consiste à chercher l’information dans des sources fiables. La pornographie et les médias montrent des hommes au sexe surdimensionné (hors-normes), ce qui motive les gens à chercher des solutions pour l’agrandissement du pénis. L'information du public par des professionnels avertis pourront éviter les frustrations surtout chez les plus jeunes. La légende des africains sur les longs pénis n’est pas scientifique, il faut mener des études scientifiques pour les prouver. L’africain et le Congolais en particulier est un homme aux dimensions du pénis comme les autres, les sources d’information doivent se méfier des déclarations non fondées sur des évidences scientifiques.

### Etat des connaissances actuelles sur le sujet

- La taille normale du pénis est connue;
- Il existe un consensus des scientifiques sur la technique de la mesure du pénis;
- Très peu d’hommes sont concernés par le micro pénis.

### Contribution de notre étude à la connaissance

- Le manque de précision dans les chiffres annoncés en RD Congo;
- L’ignorance de la technique de la mesure du pénis chez les informateurs dans notre milieu;
- Le manque d’expertise dans le domaine de la sexologie dans notre milieu.

## References

[1] Tiggemann M, Martins Y, Churchett L (2008). Beyond muscles: unexplored parts of men's body image. Journal of Health Psychology..

[2] Prause N (2015). Women's preferences for penis Size: a new research method using selection among 3D models. PLoS One.

[3] Herbenick D (2013). The Development and Validation of the Male Genital Self-Image Scale: Results from a Nationally Representative Probability Sample of Men in the United States. The Journal Of Sexual Medicine..

[4] Althof S (2003). Treatment responsiveness of the Self-Esteem And Relationship questionnaire in erectile dysfunction. Urology..

[5] Khan S (2012). Establishing a reference range for penile length in Caucasian British men: a prospective study of 609 men. BJU International.

[6] Gebhard P, Johnson A (1979). The Kinsey data: marginal tabulations of the 1938–1963 interviews.

[7] Veale D (2015). Am I normal? A systematic review and construction of nomograms for flaccid and erect penis length and circumference in up to 15,521 men. BJU Int.

[8] Dillon B, Chama N, Honig (2008). Penile size and penile enlargement surgery: a review. Int J Impot Res..

